# Tomato Prenylated RAB Acceptor Protein 1 Modulates Trafficking and Degradation of the Pattern Recognition Receptor LeEIX2, Affecting the Innate Immune Response

**DOI:** 10.3389/fpls.2018.00257

**Published:** 2018-03-01

**Authors:** Lorena Pizarro, Meirav Leibman-Markus, Silvia Schuster, Maya Bar, Tal Meltz, Adi Avni

**Affiliations:** ^1^School of Plant Sciences and Food Security, Tel Aviv University, Tel Aviv, Israel; ^2^Department of Plant Pathology and Weed Research, Agricultural Research Organization, Volcani Center, Rishon LeZion, Israel

**Keywords:** MAMP/PAMP, immunity, intracellular trafficking, degradation, SlPRA1A, PRR

## Abstract

Plants recognize microbial/pathogen associated molecular patterns (MAMP/PAMP) through pattern recognition receptors (PRRs) triggering an immune response against pathogen progression. MAMP/PAMP triggered immune response requires PRR endocytosis and trafficking for proper deployment. LeEIX2 is a well-known *Solanum lycopersicum* RLP-PRR, able to recognize and respond to the fungal MAMP/PAMP ethylene-inducing xylanase (EIX), and its function is highly dependent on intracellular trafficking. Identifying protein machinery components regulating LeEIX2 intracellular trafficking is crucial to our understanding of LeEIX2 mediated immune responses. In this work, we identified a novel trafficking protein, SlPRA1A, a predicted regulator of RAB, as an interactor of LeEIX2. Overexpression of SlPRA1A strongly decreases LeEIX2 endosomal localization, as well as LeEIX2 protein levels. Accordingly, the innate immune responses to EIX are markedly reduced by SlPRA1A overexpression, presumably due to a decreased LeEIX2 availability. Studies into the role of SlPRA1A in LeEIX2 trafficking revealed that LeEIX2 localization in multivesicular bodies/late endosomes is augmented by SlPRA1A. Furthermore, inhibiting vacuolar function prevents the LeEIX2 protein level reduction mediated by SlPRA1A, suggesting that SlPRA1A may redirect LeEIX2 trafficking to the vacuole for degradation. Interestingly, SlPRA1A overexpression reduces the amount of several RLP-PRRs, but does not affect the protein level of receptor-like kinase PRRs, suggesting a specific role of SlPRA1A in RLP-PRR trafficking and degradation.

## Introduction

Pattern recognition receptors (PRRs) are cell surface receptors that recognize pathogen/microbe-associated molecular patterns (PAMPs/MAMPs), transducing their signal and initiating innate immune responses (Jones and Dangl, 2006; Thomma et al., 2011; Newman et al., 2013). PAMP/MAMP triggered immunity (PTI/MTI) includes diverse physiological processes such as: oxidative burst, extracellular alkalinization, Ca^2+^ increase, kinase cascade activation, and callose deposition (Dodds and Rathjen, 2010; Wu et al., 2014).

Intracellular trafficking of PRRs through the endomembrane system, and particularly via endocytosis, has emerged as a requirement for PTI/MTI. PRR endocytosis is activated after PAMP/MAMP immune elicitation, and in several cases, it is necessary for triggering of defense responses (Sharfman et al., 2011; Leborgne-Castel and Bouhidel, 2014; Mbengue et al., 2016; Zarsky, 2016; Gu et al., 2017). Intracellular trafficking also plays an important role in delivering and sorting of PRRs from the endoplasmic reticulum (ER) to the plasma membrane (Lee H.Y. et al., 2011; Lefebvre et al., 2012; Choi et al., 2013). In addition, after PAMP/MAMP recognition, PRRs can traffic toward the vacuole for degradation, resulting in signal attenuation (Lu et al., 2011; Spallek et al., 2013; Smith et al., 2014; Mbengue et al., 2016; Ortiz-Morea et al., 2016).

The machinery regulating intracellular trafficking includes RAB small GTPases (Stenmark, 2009; Mizuno-Yamasaki et al., 2012; Pizarro and Norambuena, 2014). RABs are located in specific cellular compartments and play important roles in the reception and selection of the vesicles when reaching a target compartment (Bonifacino and Glick, 2004; Pfeffer, 2013). RABs can act as molecular switches, being activated and inactivated through a set of regulators, including GTPase activating protein (GAP), guanosine nucleotide dissociation inhibitors (GDI), and guanine nucleotide exchange factors (GEF) (Stenmark, 2009). Interestingly, RAB proteins regulate intracellular trafficking during defense responses, and have been implicated in pathogen response deployment. For example, RAB11/RABA subgroup regulates FLS2-PRR trafficking, particularly mediating exocytosis and PRR delivery to the plasma membrane (Choi et al., 2013). In rice, OsRAB11enhances resistance to *Pseudomonas syringae* and in Arabidopsis, AtRABG3b promotes cell death and hypersensitive response to *P. syringae* (Kwon et al., 2013).

The fungal protein ethylene-inducing xylanase (EIX) (Dean et al., 1989) is a well-known elicitor of defense responses in tobacco (*Nicotiana tabacum*) and tomato (*Solanum lycopersicum*) (Bailey et al., 1990; Avni et al., 1994; Ron et al., 2000). EIX induces ethylene biosynthesis, oxidative burst, media alkalinization, expression of pathogen related (PR) proteins and hypersensitive response in responsive plant species and/or varieties (Bailey et al., 1990, 1993; Ron et al., 2000; Elbaz et al., 2002; Laxalt et al., 2007). The response to EIX is controlled by *LeEIX2*, which encodes a PRR type receptor-like protein (RLP) (Ron and Avni, 2004). We have previously shown that intracellular trafficking is crucial for triggering EIX-induced responses (Bar and Avni, 2009; Bar et al., 2009; Sharfman et al., 2011). Following exposure to EIX, LeEIX2 is internalized from the plasma membrane to late endosomes via clathrin mediated endocytosis (Sharfman et al., 2011). Consequently, chemical and genetic inhibition of LeEIX2 endocytosis strongly compromises EIX-induced response (Sharfman et al., 2011) highlighting the relevance of LeEIX2 trafficking in the EIX defense response.

The machinery regulating LeEIX2 trafficking from the ER to the plasma membrane and the vacuole remains unclear. We undertook a split ubiquitin yeast two-hybrid (Y2H) screen to identify trafficking machinery components interacting with LeEIX2. This screen revealed a prenylated RAB acceptor protein type 1 ortholog from *S. lycopersicum* (SlPRA1A). Prenylated RAB acceptor proteins of type1 are transmembrane proteins present in animals, fungi and plants, which participate in the regulation of RAB proteins (Hutt et al., 2000; Figueroa et al., 2001; Bahk et al., 2009). In this work, we present evidence showing that SlPRA1A regulates LeEIX2 intracellular trafficking, possibly by driving it to degradation, subsequently decreasing EIX-induced responses.

## Materials and Methods

### Plant Growth Conditions

*Nicotiana tabacum* cv. samsun NN and *N. benthamiana* plants were grown from seeds in a greenhouse under long day conditions (16 h light and 8 h dark) at 24°C.

### Plasmid Construction

For overexpression assays, *SlPRA1A* cDNA (Solyc03g121460) C-terminally tagged with GFP, mCherry or 2xHA was cloned into pBINPLUS (van Engelen et al., 1995) using the following primers: SlPRA1A forward primer 5′-TAGTCGACATGACGAATTACGGCACAATACC-3′ and SlPRA1A reverse primer 5′-TAGGATCCAGACGACGGAGCAGAAG-3′, between the CAM35SΩ promoter containing the translation enhancer signal and the NOS terminator. SlPRA1A N62T and Y70A single mutations were generated sequentially using Q5^®^ Site-Directed Mutagenesis Kit (New England Biolabs). Primers were designed using the NEBaseChanger tool from the New England Biolabs inc. website. The N62T mutation was generated using the following primers: forward primer 5′-AATCAAGACAACTTTCTCCTTCTTC -3′ and reverse primer 5′-CGTGATATAGCATCGCTG -3′. The Y70A mutation was generated using the following primers: forward primer 5′-CCAGACGAACGCCGCCATCATAGTG -3′ and reverse primer 5′-AAGAAGGAGAAAGTTGTC -3′. The mutations were verified by sequencing and the mutated SlPRA1 was cloned into pBINPLUS (van Engelen et al., 1995) for tagging with GFP or mCherry. *LeEIX2* cDNA (Solyc07g008630) C-terminally tagged with GFP was cloned into the *Sal*I site of pBINPLUS (van Engelen et al., 1995) using the following primers: *LeEIX2* forward primer 5′-ATGTCGACATGGGCAAAAGAACTAATC-3′ and *LeEIX2* reverse primer 5′-ATGTCGACGTTCCTTAGCTTTCCCTTCAGTC-3′.

### Co-immunoprecipitation

Co-immunoprecipitation assays were performed as previously described (Leibman-Markus et al., 2017). Briefly, *N. benthamiana* leaves transiently co-expressing either LeEIX2 or GFP tagged LeEIX2 and either mCherry tagged SlPRA1A or free mCherry were harvested 40 h after infiltration. Leaf petioles were immersed in EIX 300 μg/mL (or water as mock) for 7.5 min and then transferred to water for an additional 7.5 min. 300 mg tissue from each treatment was used for co-immunoprecipitation as described (Leibman-Markus et al., 2017), using 8 μl GFP-TrapA beads incubated in Immunoprecipitation buffer [50 mM Tris-HCl, pH 7.5, 150 mM NaCl, 6 mM β-mercaptoethanol, 0.5%TritonX-100, 1X protease inhibitor cocktail (EDTA free) at 4°C for 4 h (Chromotek, Planegg-Martinsried, Germany)].

### Yeast Two Hybrid Screening

Yeast two-hybrid analyses were performed essentially as described in the DUALmembrane kit user manual (Dualsystems Biotech AG). *LeEIX2* (Solyc07g008630) without the signal peptide region was cloned into the “bait” plasmid pBT3SUC using the following primers: LeEIX2 forward primer 5′-ATTAAAAAGGCCATTACGGCCTTAACTTCAAGAGAAGTTAAC-3′, LeEIX2 reverse primer 5′-AACTGATTGGCCGAGGCGGCCCCGTTCCTTAGCTTTCCCTTCAG-3′. A *S. lycopersicum* cDNA library was cloned into the “prey” plasmid pPR3N. To carry out the Y2H screen, the cDNA library was transformed into the yeast strain NMY51 carrying the pBT3SUC-LeEIX2 plasmid. Selection of interacting proteins was performed based on β-galactosidase activity in auxotrophic medium (Leu^-^ and Trp^-^), for the selection of both plasmids, supplemented with *X*-Gal (0.1 mg/mL).

### Transient Expression by Agroinfiltration

Binary vector clones were introduced into *Agrobacterium tumefaciens* strain GV3101 by electroporation. Agrobacterium cells were grown in LB medium containing 50 mg/L Kanamycin, 40 mg/L Gentamicin and 100 mg/L Rifampicin overnight at 28°C, diluted into VIR induction medium [50 mM MES pH 5.6, 0.5% (w/v) glucose, 1.7 mM NaH_2_PO4, 20 mM NH_4_Cl, 1.2 mM MgSO_4_, 2 mM KCl, 17 μM FeSO_4_, 70 μM CaCl_2_ and 200 μM acetosyringone] and grown for six additional hours until OD_600_ reached 0.4–0.6. Single or mixed Agrobacterium cultures were then diluted to a final OD_600_ of 0.15 each in VIR induction medium. Diluted Agrobacterium cultures were infiltrated into leaves of *N. tabacum* cv. samsun NN or *N. benthamiana* using a needless syringe. Experiments were performed 40 h after infiltration, unless otherwise specified.

### EIX Purification

Ethylene-inducing xylanase was purified from a crude extract of xylanase from *Trichoderma viride* (Sigma–Aldrich CAS Number 9025-57-4) following the described purification protocol (Dean and Anderson, 1991).

### ROS Measurement

Reactive oxygen species (ROS) burst was measured as previously described (Leibman-Markus et al., 2017). Leaf disks 0.5 cm in diameter were taken from transiently expressing plants 40 h post infiltration. Disks were floated in a white 96-well multiplate containing 250 μl ddH_2_O for 4–6 h at room temperature. After incubation, water was removed, ROS measurement reaction containing either 1 μg/mL EIX, 1 mM Flg22 or water as mock was added and light emission was immediately measured every 3.5 min using a Turner biosystems Veritas Luminometer.

### Ethylene Measurement

Ethylene biosynthesis was measured as previously described (Leibman-Markus et al., 2017). Leaf disks 0.9 cm in diameter were taken from transiently expressing plants 40 h post-infection. Every five disks were sealed in a 10 mL flask containing 1 ml assay medium (with or without 1 μg/mL EIX) for 4 h at room temperature. Ethylene production was measured by gas chromatography using Varian 3350 equipment.

### Immunoblotting

One hundred mg of *N. benthamiana* leaves transiently expressing relevant proteins were ground to a fine powder with liquid nitrogen and 2.5 volumes of extraction buffer (50 mM Tris-HCl, pH 7.5, 2 mM MgCl_2_, 150 mM NaCl, 1% triton x-100, 140 mM β-mercaptoethanol, 2 mM PMSF and 1 mM EDTA-free protease inhibitor cocktail) were added. Samples were incubated in a rotating wheel at 4°C for 20 min before centrifugation. Supernatant samples were collected and boiled after adding sample buffer [8% SDS, 40% glycerol, 200 mM Tris-Cl, pH 6.8, 388 mM dithiothreitol (DTT), and 0.1 mg/ml bromophenol blue dye]. Samples were run in SDS-PAGE and blotted onto nitrocellulose membranes. The following primary antibodies were used: mouse α-mCherry (Chromotek), rat α-GFP (Chromotek) and rabbit α-Luciferase (Sigma–Aldrich).

### Confocal Microscopy

**FIGURE 1:**
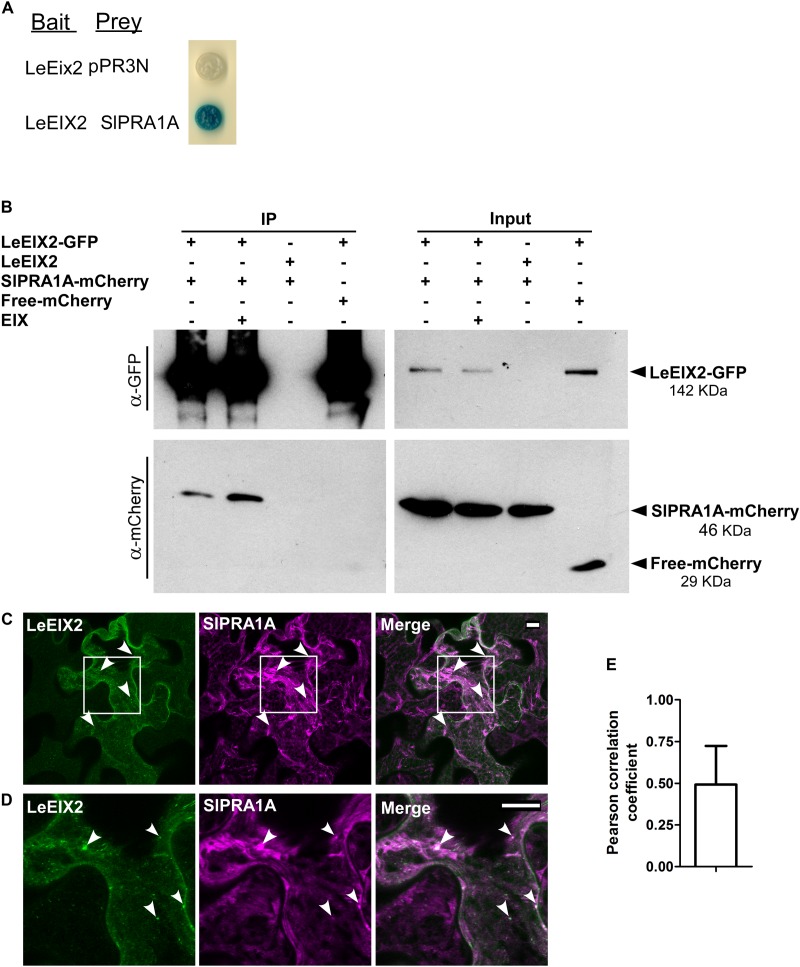
SlPRA1A, a *Solanum lycopersicum* prenylated RAB acceptor 1, associates with the PRR LeEIX2. **(A)** Identification of SlPRA1A through split-ubiquitin yeast-two hybrid. NMY51 yeast cells containing LeEix2 (in pBT3SUC, Bait), SlPRA1A (in pPR3N, Prey) or LeEix2 (in pBT3SUC) and empty vector pPR3N were grown on galactose medium lacking the relevant amino acids and supplemented with *X*-gal. **(B)** Co-immunoprecipitation assays *in planta* in the presence or absence of EIX were performed in *Nicotiana benthamiana*. Non-tagged LeEIX2 was used as a control for immunoprecipitation, and free-mCherry was used as a control for interaction specificity Representative results of four independent experimental replicates are shown. **(C,D)** Confocal microscopy images of *N. benthamiana* epidermal cells transiently expressing LeEIX2-GFP (green) and SlPRA1A-mCherry (magenta). **(D)** Representative maximum Z-stack projection images are shown. Scale bar 10 μm. **(E)** Pearson correlation coefficient of SlPRA1A and LeEIX2 co-localization was determined using Coloc2 from FIJI-ImageJ, using sixteen images. Data represented as mean ± SD.

**FIGURE 2:**
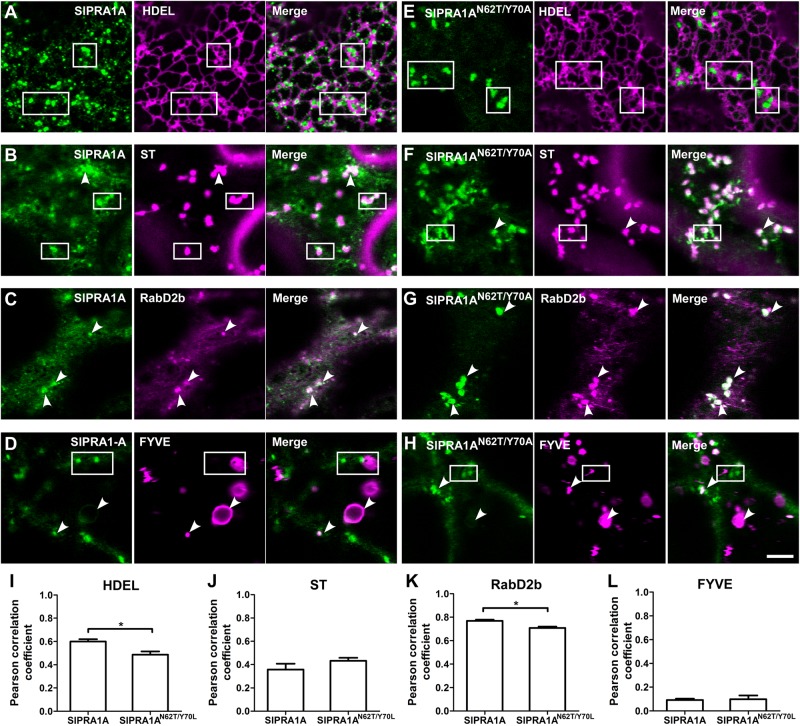
SlPRA1A is localized in RabD2b-TGN/EE adjacent to the ER. **(A–H)** Confocal microscopy images of *N. benthamiana* epidermal cells transiently expressing SlPRA1A-GFP or SlPRA1A^*N62T/Y 70A*^ -GFP and different endomembrane compartment markers as indicated. Representative images show SlPRA1A-GFP or SlPRA1A^*N62T/Y 70A*^-GFP (green), the compartment marker (magenta) and the superimposed image of both channels (merge). **(A,D)** SlPRA1A. **(E,H)** SlPRA1A^*N62T/Y 70A*^. **(A,E)** The HDEL ER marker fused to mCherry. **(B,F)** The Sialyltransferase (ST) Golgi marker fused to mCherry. **(C,G)** The RabD2b-TGN/EE marker fused to mCherry. **(D,H)** The FYVE late endosome marker fused to dsRed. Scale bar 5 μm. White arrowheads point to SlPRA1A compartments co-localizing with the marker. Squares mark areas where SlPRA1A compartments and the marker do not co-localize. **(I–L)** Pearson correlation coefficient of the co-localization between SlPRA1A and the markers (*N* = 20–30). Data represented as mean ± SEM. Asterisks represent statistical significance (*p*-value ≤ 0.05) in a student’s *t*-test.

Confocal microscopy images were acquired using a Zeiss LSM780 confocal microscope system with Objective LD SC Plan-Apochromat 20×/1.0 Corr M32 (**Figure 5F**), Objective C-Apochromat 40×/1.2 W Corr M27 (**Figures 1**, **2**, **5A–D**, **6**, **7**) or Objective C-Apochromat 63×/1.2 W Corr (**Figure 4**). Acquisition settings were designed using two tracks. Track 1 collected the chlorophyll fluorescence using an excitation laser wavelength of 633 nm (2% power). The emission was then collected in the range of 652–721 nm. Track 2 used two different channels to collect GFP and dsRed or mCherry fluorescence using an excitation laser of 488 nm (5% power) and 561 nm (3% power), respectively. For GFP, emission was collected in the range of 493–535 nm, and for dsRed or mCherry, emission was collected in the range of 588–641. Images of 8 bits and 1024 × 1024 were acquired using a pixel dwell time of 1.27, pixel averaging of 4 and pinhole of 1 airy unit. Z-sections of 2 μM were made ensuring a 50% overlap of each slice in the Z-stack. Image analysis was performed using Fiji-ImageJ with the raw images (Schindelin et al., 2012). Co-localization analysis was performed with the Coloc2 tool, endosome count and size measurements were done with the 3D Object counter tool, pixel intensity was measured using the measurement analysis tool and the heat map was generated with the Heatmap histogram plugin.

### Concanamycin A Treatment

*Nicotiana benthamiana* leaves were infiltrated with a water solution of ConcA 2 μM (Stock solution 1 mM in DMSO, CAS 80890-47-7, Santa Cruz) or a water solution containing an equivalent amount of DMSO (Mock, DMSO 0.2%), using a needless syringe, 16 h after agroinfiltration. 24 h after infiltration the ConcA solution and Mock solution were applied to the leaves through the petiole. The tissue was collected 40 h after agroinfiltration, completing 24 h of ConcA treatment.

### RNA Extraction and qRT-PCR Analysis

Plant total RNA was extracted using SV Total RNA Isolation System (Promega, Madison, WI, United States). 4 μg RNA samples were subjected to first strand cDNA synthesis using M-MLV reverse transcriptase (Promega, Madison, WI, United States) and oligodT15. qRT-PCR was performed according to the Fast SYBR Green Master Mix protocol (Life Technologies, Thermo Fisher, Waltham, MA, United States), using a StepOnePlus machine (Thermo Fisher, Waltham, MA, United States). RT-qPCR was performed using the following cycles; 95°C for 10 min, followed by 40 cycles of 95°C for 15 s and 60°C for 45 s. Control samples without reverse transcriptase did not generate a PCR product after 38 amplification cycles, indicating the samples were free of genomic DNA contamination. LeEIX2 expression (Solyc07g008630) was examined using forward primer 5′-ACCAGGAGTCCGAGTACAAGA-3′ and reverse primer 5′- TGACAAGTCGAGGGACTCCA-3′. Endogenous reference gene Ubi3 from *N. benthamiana* was amplified using 5′- AATGTGAAAGCCAAGATCCAAG-3′ and reverse primer 5′- CGGAGGCGGAGCACGAGATGAA-3′ (Liu et al., 2012).

### Phylogenetic Tree

Phylogenetic analysis of SlPRA1A (Solyc03g121460) was performed using the PRA1 protein sequences from *Arabidopsis thaliana, S. lycopersicum*, and *Oryza sativa*, and protein sequences of PRA1 from *Mus musculus* (Mouse), *Homo sapiens* (Human), and *Saccharomyces cerevisiae* (Yeast). The sequences of PRA1 proteins from *S. lycopersicum* and *O. sativa* were searched by BLAST in Sol Genomic Network and Rice Annotation Project, using all the *A. thaliana* PRA1 sequences. A maximum likelihood phylogenetic tree was generated through the software http://phylogeny.lirmm.fr/phylo_cgi/phylogeny.cgi, using the following pipeline: MUSCLE for multiple alignment, PhyML for tree building, and TreeDyn for tree rendering (Dereeper et al., 2008). Bootstrapping of 1000 was used.

### Accession Numbers

At5g02040, At5g05987, At3g11397, At3g56110, At2g40380, At5g05380, At2g38360, At5g01640, At5g01640, At4g29658, At1g04260, At1g08770, At1t17700, At1g55190, At3g13720, At3g13710, At1g55640, At5g56230, AT4G27540, Solyc09g011310, Solyc10g084920, Solyc09g015155, Solyc10g076360, Solyc06g050570, Solyc09g083320, Solyc07g066190, Solyc09g066000, Solyc03g121460, Solyc01g097230, Solyc12g013910, Solyc07g008630, Os05g38160, Os01g10010, Os01g62890, Os01g62890, Os12g01690, Os05g11120, Os03g53070, Os05g39670, Os01g61170, Os11g01610, Os07g41940, Os03g58410, Os09g04880, Os01g10010, Os10g41420.

## Results

### Identification of SlPRA1A, a Prenylated RAB Acceptor 1 Protein, as a Novel LeEIX2 Interactor

To identify components involved in intracellular trafficking of the LeEIX2 receptor (Ron and Avni, 2004), a split ubiquitin Y2H screen was performed. The cDNA encoding LeEIX2 (Solyc07g008630) was fused to the C-terminal half of ubiquitin in the “bait” plasmid, and a *S. lycopersicum* cDNA library was cloned into the prey plasmid (Dualsystems Biotech). We screened approximately 1 × 10^6^ independent clones of the *S. lycopersicum* cDNA library. Positive clones for LeEIX2 interaction (**Figure 1A**) were isolated and sequenced. Phylogenetic analysis of one of the isolated clones identified Solyc03g121460, a *S. lycopersicum* prenylated RAB acceptor 1 homolog, termed SlPRA1A (Supplementary Figure S1), as an interactor of LeEIX2. PRA1 proteins are transmembrane proteins that regulate RAB function. Specifically, PRA1 proteins stabilize RAB proteins at cell membranes, thus activating RABs and promoting trafficking (Hutt et al., 2000; Figueroa et al., 2001; Bahk et al., 2009).

LeEIX2 and SlPRA1A were transiently overexpressed in *N. benthamiana* and the interaction between them was verified *in planta* by co-immunoprecipitation (Co-IP). We immunoprecipitated GFP-LeEIX2, leading to a strong enrichment of this protein after immunoprecipitation (**Figure 1B**). SlPRA1A was successfully pulled down together with LeEIX2 (**Figure 1B**), confirming the split ubiquitin Y2H data. The Co-IP assays were performed in the presence and absence of EIX. SlPRA1A was pulled down with LeEIX2 in both cases, showing that EIX exposure is not required for the interaction (**Figure 1B**). EIX elicitation may stimulate the interaction between SlPRA1A and LeEIX2, as SlPRA1A pull down was slightly stronger after EIX treatment. Through live cell imaging, we observed that SlPRA1A is localized in a reticular and punctuated pattern at the subcellular level, partially co-localized with the LeEIX2 receptor having a Pearson correlation coefficient of 0.49 ± 0.22 (**Figures 1C–E**), providing the subcellular context where both proteins can interact.

### SlPRA1A Is Located at a RabD2b-Positive *Trans*-Golgi Network/Early Endosome (TGN/EE) Compartment Surrounding the ER

SlPRA1A is a transmembrane protein with four predicted transmembrane domains, according to TMHMM and TOPCONS transmembrane predictor methods (Moller et al., 2001; Tsirigos et al., 2015). SlPRA1A, as was mentioned above, is a predicted PRA1 proteins, thus is expected to be located in the endomembrane system, similar to from other organisms (Lin et al., 2001; Sivars et al., 2005; Alvim Kamei et al., 2008). In *A. thaliana* PRA1 proteins has been classified on eight groups the localize in different compartments within the endomembrane system (Alvim Kamei et al., 2008). Between them, AtPRA1.B6, AtPRA1.F4 and a rice ortholog from the B-type clade OsPRA1 (Os05g39670, Supplementary Figure S2) has been characterized as trafficking regulators that are localized in the endoplasmic reticulum (ER) or near by the ER on endosomal and Golgi compartments (Heo et al., 2010; Lee M.H. et al. 2011, 2017). Live cell imaging confocal microscopy was used to further determine SlPRA1A subcellular localization co-expressing SlPRA1A with different endomembrane marker. SlPRA1A partially co-localized with the HDEL ER marker (Nelson et al., 2007) (Pearson correlation coefficient of 0.60 ± 0.08; **Figures 2A,I**). Indeed, SlPRA1A was observed in a fuzzy reticular pattern and also localized in some discrete punctuated structures surrounding the ER (**Figure 2A**). In order to determine the identity of the SlPRA1A punctuated compartment, we used the Sialyltransferase and GmMan1, soybean α-1,2-mannosidase I Golgi markers and RabD2b and VHAa1 *trans*-Golgi network/Early endosome (TGN/EE) markers (Saint-Jore-Dupas et al., 2006; Nelson et al., 2007; Geldner et al., 2009; Ivanov and Harrison, 2014). SlPRA1A minimally co-localized with Golgi bodies, (Pearson correlation coefficient of 0.36 ± 0.17 and 0.41 ± 0.16, respectively) (**Figures 2B,J** and Supplementary Figures S2A,B) and highly co-localized with the RabD2b TGN/EE marker (Pearson correlation coefficient of 0.77 ± 0.03) (**Figures 2C,K**) but not the VHAa1 marker (Pearson correlation coefficient of 0.55 ± 0.13) (Supplementary Figures S2C,D). In addition, we tested the co-localization between SlPRA1A and the FYVE protein marker which interacts with Inositol 3 phosphate membranes typically found in multi vesicular body/late endosomes (MVB/LE) (Vermeer et al., 2006; Simon et al., 2014). Although SlPRA1A1 was absent from most FYVE compartments (Pearson correlation coefficient of 0.10 ± 0.04; **Figures 2D,L**), it was identified in specific large MVB/LE FYVE-positive organelles (**Figure 2D**). It is important to note that RabD2b is not a canonical TGN/EE marker, it has also been described as a protein localized in endosomal/Golgi compartments (Pinheiro et al., 2009; Peng et al., 2011), in accordance with the partial colocalization observed between SlPRA1A and Golgi marker. Overall, our analyses indicate that SlPRA1A is localized primarily in RabD2b compartments, surrounding the ER, in a punctuate-reticular pattern. Concomitantly, the RabD2b compartment size decreased upon SlPRA1A overexpression, suggesting that SlPRA1A function is related to this compartment, which may depend on SlPRA1A1 for its maintenance and/or function (Supplementary Figures S3A,B).

The roles of mammalian and yeast PRA1 proteins have been previously examined via mutational analysis. Mutations proximal to the first hydrophobic domain of human HsPRA1 modify its subcellular localization and abolish its ability to interact with HsRAB3A, leading to loss of its function (Gougeon et al., 2002). Analogous mutations were generated in rice OsPRA1, impairing its capability to bind OsRAB7, resulting in inhibition of vacuolar trafficking (Heo et al., 2010). We used site directed mutagenesis to generate a loss of function mutant of SlPRA1A, mutating the analogous residues in the first hydrophobic domain of SlPRA1A (N62T and Y70A). In order to determine if the function of SlPRA1A^*N62T/Y 70A*^ had altered, we analyzed its subcellular localization and co-localization with the endosomal markers. SlPRA1A^*N62T/Y 70A*^ was mainly located in discrete compartments, which are larger than the wild type SlPRA1A compartments (**Figures 2E–H** and Supplementary Figure S3C). In spite of the localization of SlPRA1A^*N62T/Y 70A*^ in larger RabD2b compartments, the general colocalization between them was decreased as compared to the wild-type protein (**Figure 2K**). Furthermore, the localization of SlPRA1A^*N62T/Y 70A*^ at the ER was decreased as compared to the wild-type protein, and this may explain the decrease in general colocalization (**Figure 2I**). Additionally, no difference in Golgi and FYVE compartment localization was observed between SlPRA1A^*N62T/Y 70A*^ and SlPRA1A (**Figures 2H,L**). Notably, reduction of the RabD2b compartments size was not observed upon SlPRA1A^*N62T/Y 70A*^ overexpression (Supplementary Figures S3A,B). This observation together with the reduced colocalization with the ER-marker and RabD2b indicates that SlPRA1A^*N62T/Y 70A*^ may have diminished functionality.

### SlPRA1A Overexpression Impairs the Physiological Response to EIX in *N. tabacum* and *S. lycopersicum*

**FIGURE 3:**
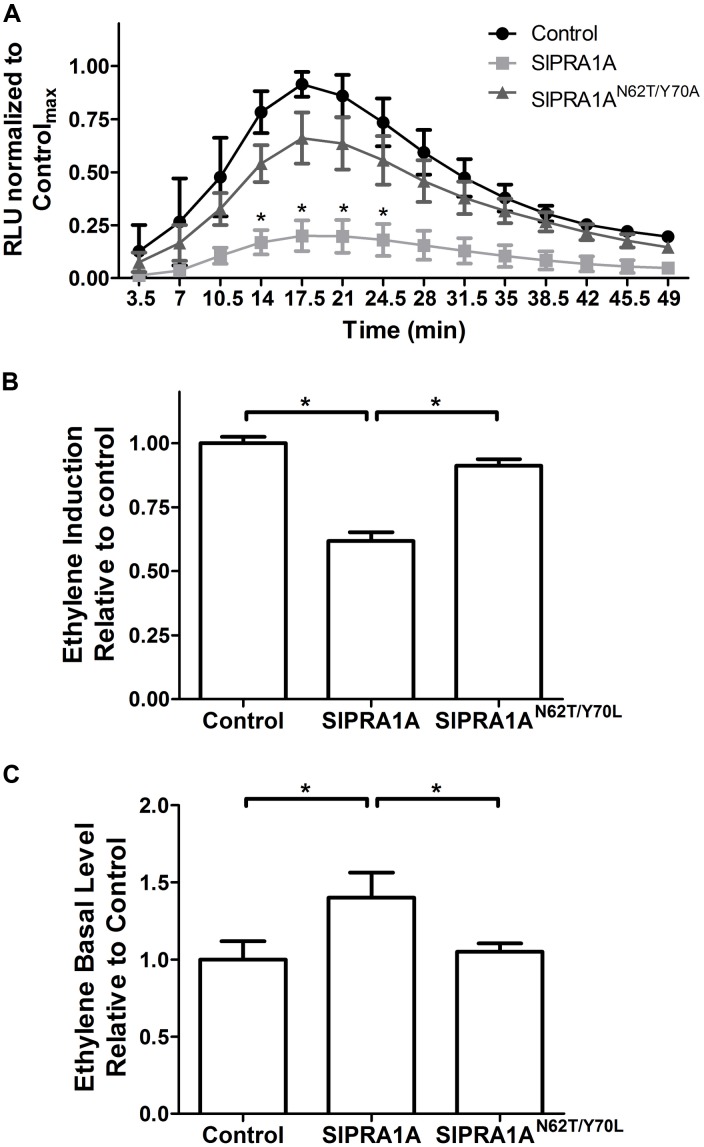
SlPRA1A overexpression attenuates EIX induced defense responses in *N. tabacum*. **(A)** ROS oxidative burst was measured in *N. tabacum* transiently expressing free mCherry (control), SlPRA1A-mCherry or SlPRA1A^*N62T/Y 70A*^-mCherry using a luminol luminescence based system. ROS production is normalized to the control peak value in each experiment. Average and standard deviation of four independent replicates are shown. Asterisks represent statistical significance (*p*-value ≤ 0.05) in two-way ANOVA and Bonferroni post-tests. Data are represented as mean ± SEM. **(B,C)** Ethylene was measured by gas-chromatography in *N. tabacum* transiently expressing free-mCherry (control), SlPRA1A-mCherry or SlPRA1A^*N62T/Y 70A*^-mCherry treated with EIX or mock (water) for 4 h. **(B)** Induction of ethylene production after EIX exposure was calculated as the ratio between ethylene level after EIX exposure and mock treatment, which was then normalized to control. **(C)** Ethylene production in mock treatment (basal level) is presented as the ratio to control. Average and standard deviation of four independent experiments are shown. Data are presented as mean ± SEM. Asterisks represent statistical significance (*p*-value ≤ 0.05) in one-way ANOVA and Tukey post-tests.

Ethylene-inducing xylanase induces a variety of defense responses in sensitive species, such as *N. tabacum* and *S. lycopersicum*, including oxidative burst and induction of ethylene production (Laxalt et al., 2007; Bar and Avni, 2009). We examined the effect of SlPRA1A on these two EIX responses. The oxidative burst induced by EIX in *N. tabacum* peaks approximately 20 min after EIX exposure (**Figure 3A**). SlPRA1A overexpression strongly inhibited the oxidative burst induced by EIX, causing a decrease of over 80% in ROS production compared to the control (**Figure 3A** and Supplementary Figure S4A). In agreement, SlPRA1A overexpression also reduced the induction of ethylene production triggered by EIX by ∼35% as compared to the control (**Figure 3B** and Supplementary Figure S4B). The SlPRA1A^*N62T/Y 70A*^ mutant did not impair the oxidative burst (**Figure 3A**) or the ethylene biosynthesis (**Figure 3B**), behaving similarly to the control. These results led us to conclude that SlPRA1A impairs plant responses to EIX. Intriguingly, ethylene production was induced to a certain extent without EIX exposure (**Figure 3C**), suggesting a possible link between SlPRA1A and ethylene production.

**FIGURE 4:**
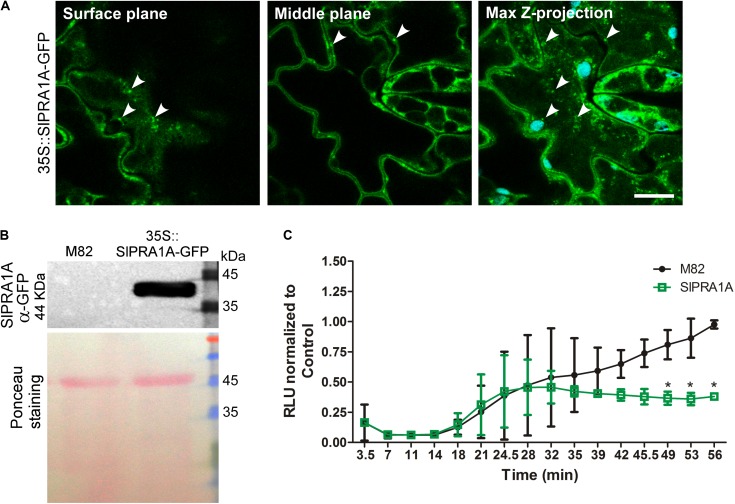
Effect of SlPRA1A overexpression on EIX response in *S. lycopersicum*. **(A)** Subcellular localization of SlPRA1A-GFP expressed in 35S::SlPRA1A-GFP transgenic line. Representative images of surface cell plane, middle cell plane and maximum Z-stack projection are showed. White arrowheads point to SlPRA1A compartments. Scale bar 10 μm. **(B)** Total protein extraction of 35S::SlPRA1A-GFP *S. lycopersicum* transgenic stable line, following SDS-PAGE and western blotting were performed to determine SlPRA1A- GFP expression. **(C)** ROS oxidative burst was measured through a luminol luminescence based system using *S. lycopersicum* M82 ecotype (background line, control) and 35S::SlPRA1A-GFP line. ROS production was normalized to the control peak value in each experiment. Average and standard deviation of four independent experimental replicates are shown. Data are represented as mean ± SD. Asterisks represent statistical significance (*p*-value ≤ 0.05) in two-way ANOVA and Bonferroni post-test.

In order to test the role of SlPRA1A in *S. lycopersicum* we generated a transgenic line expressing SlPRA1A fused to GFP driven by the 35S CaMV promoter over the M82 background ecotype (**Figures 4A,B**). We observed that SlPRA1A is localized in a punctuated pattern in epidermal cells of *S. lycopersicum* leaf (**Figure 4A**). This pattern resembles the punctuated distribution observed in SlPRA1A transiently expressed in *N. benthamiana*, although the reticular pattern was not observed. Additionally, responsiveness to EIX was evaluated in the SlPRA1A overexpressing stable line through ROS measurement. The oxidative burst triggered by EIX reaches its peak 1 h after EIX exposure in M82 ecotype (**Figures 3A**, **4C**). Differences between *S. lycopersicum* and *N. benthamiana* in amplitude and response time to EIX could be due to the overexpression method, stable or transient, respectively, or to differences in the basal response levels characteristic of the different species (**Figure 4C**). In the SlPRA1A transgenic line the oxidative burst decreased strongly, over 60% compared to the background ecotype (**Figure 4C**), supporting SlPRA1A role on EIX response inhibition in *S. lycopersicum*.

### SlPRA1A Overexpression Decreases LeEIX2 Protein Levels

**FIGURE 5:**
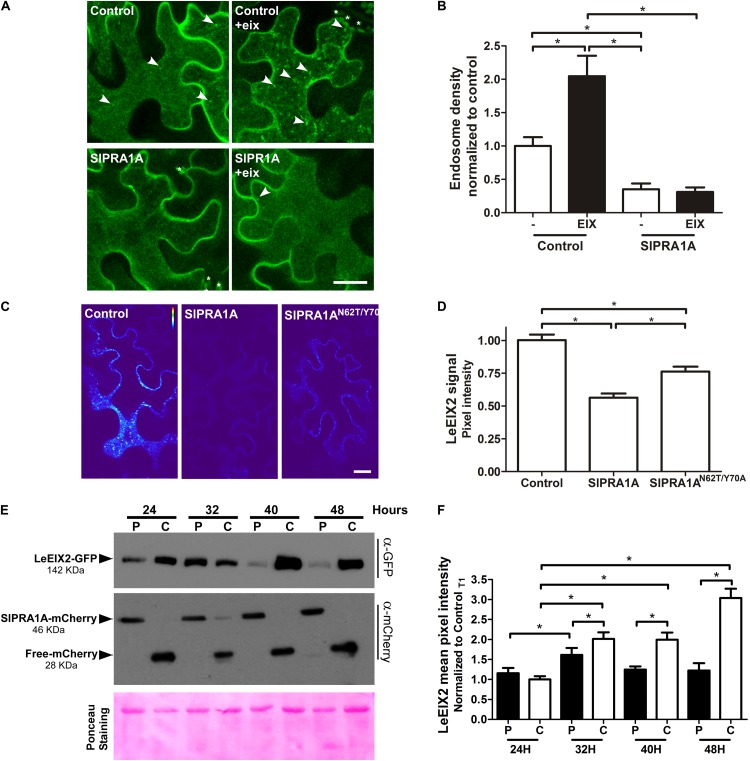
SlPRA1A overexpression decreases LeEIX2 endosomal localization and protein level. **(A)** Confocal microscopy images of *N. benthamiana* epidermal cells transiently expressing LeEIX2-GFP and Free-mCherry (control) or SlPRA1A-mCherry treated with EIX. Z-maximum projection of five slices was performed (2 μm) and images were analyzed using Fiji-ImageJ. Representative images from four replicates of four images are shown, contrast was adjusted uniformly in order to improve GFP channel visualization (see also Supplementary Figure S5A). Scale bar 10 μm. **(B)** LeEIX2 endosome density of the experiment showed in **(A)**. Thirty images were analyzed. Data are presented as mean ± SD. Asterisks represent statistical significance in one-way ANOVA and Tukey post-test (*p*-value ≤ 0.05). **(C)** Heat map of LeEIX2-GFP (non-contrast adjustment) co-expressed with free-mCherry (Control), SlPRA1A-mCherry or SlPRA1A^*N62T/Y 70A*^-mCherry. Z-maximum projection of five slices was performed (2 μm), representative images are shown (see also Supplementary Figure S5B). Scale bar 20 μm. **(D)** Measurement of LeEIX2-GFP mean pixel intensity (*N* = 30). Data are represented as mean ± SD Two-way ANOVA and Bonferroni post-test were performed, *p*-value ≤ 0.05. **(E)** Plant samples were collected every 8 h, between 24 and 48 h after agroinfiltration of LeEIX2-GFP with free-mCherry (C, control) or SlPRA1A-mCherry (P), The experiment was replicated four times independently. Representative images are shown. **(F)** LeEIX2-GFP was tracked by imaging every 8 h between 24 and 48 h after agroinfiltration of LeEIX2-GFP and free-mCherry (C, control) or SlPRA1A-mCherry (P) (see also Supplementary Figure S5). Data are presented as mean ± SD (*N* = 20). Asterisks represent statistical significance (*p*-value ≤ 0.05) in two-way ANOVA and Bonferroni post-test.

SlPRA1A likely affects cellular trafficking through its predicted regulation of RAB GTPases. We examined whether SlPRA1A affects EIX responses through regulation of LeEIX2 trafficking. At steady state, LeEIX2 is localized mainly at the plasma membrane and in endosomal compartments (Sharfman et al., 2011). Following induction by EIX, LeEIX2 is endocytosed, increasing its endosomal localization 2.5-fold compared to control conditions (**Figures 5A,B** and Supplementary Figure S5A) (Sharfman et al., 2011). When combined with SlPRA1A overexpression, the steady-state endosomal localization of LeEIX2 considerably decreased (**Figures 5A,B**). Concomitantly, SlPRA1A overexpression abolished EIX-induced LeEIX2 endocytosis (**Figures 5A,B** and Supplementary Figure S5A). This suggests that SlPRA1A regulates LeEIX2 trafficking, reducing LeEIX2 endosomal localization and inhibiting its endocytosis following EIX exposure. In addition, the signal intensity observed by confocal microscopy showed that LeEIX2 protein levels were strongly reduced when SlPRA1A was overexpressed (**Figures 5C,D** and Supplementary Figure S5B). Overexpression of the mutant SlPRA1A^*N62T/Y 70A*^ version resulted only in a slight reduction of LeEIX2 protein levels (**Figures 5C,D** and Supplementary Figure S5B).

To corroborate the effect of SlPRA1A on LeEIX2 protein levels, we examined the time-dependent accumulation of LeEIX2 following SlPRA1A overexpression, as compared to control conditions, using both confocal microscopy and immunoblotting (**Figures 5E,F** and Supplementary Figure S5C). LeEIX2 protein levels were sampled every 8 h over 24 h, starting 24 h after agroinfiltration. In the control, LeEIX2 started to accumulate 24 h after infiltration, reaching its maximum expression at 48 h after agroinfiltration. However, in the background of SlPRA1A overexpression, LeEIX2 was barely detectable 48 h after infiltration, showing a peak at 32 h, at considerably lower levels than the control (**Figures 5E,F**).

**FIGURE 6:**
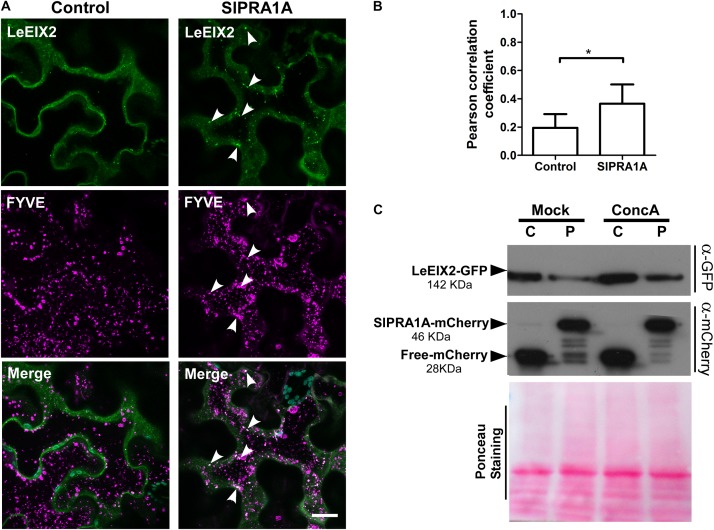
SlPRA1A overexpression decreases LeEIX2 protein level by redirecting it to vacuolar degradation. **(A)** Confocal microscopy images of *N. benthamiana* epidermal cells transiently expressing LeEIX2-GFP (green), FYVE- dsRed late endosome marker (magenta) co-expressed with Free-HA (control) or SlPRA1A-HA. Images were acquired 30 h after agroinfiltration. Arrowheads indicate endosomal structures where LeEIX2 co-localizes with the FYVE-late endosome marker. Representative images of Z-maximum projections of five slices (2 μm) are shown. Scale bar 20 μm. **(B)** Pearson correlation coefficient of the co-localization between SlPRA1A and the endosomal markers (*N* = 20). Data are presented as mean ± SEM. Asterisks represent statistical significance (*p*-value ≤ 0.05) in Student’s *t*-test. **(C)** LeEIX2 protein level in *N. benthamiana* leaves overexpressing LeEIX2-GFP and free-mCherry (C) or SlPRA1A (P) after ConcanamycinA vacuolar degradation inhibition (ConcA 2 μM, 24 h) or under control conditions (Mock). The experiment was independently replicated four times. Representative images are shown.

Considering that LeEIX2 expression was driven by the 35S CaMV promoter, showing the accumulation peak 32 h after agroinfiltration, we suggest that SlPRA1A strongly compromises LeEIX2 accumulation/degradation, but not LeEIX2 expression. In fact, no changes on LeEIX2 mRNA level were detected between all the time points analyzed on the SlPRA1 background overexpression, although LeEIX2 mRNA level was lower on the SlPRA1 background overexpression than the control (Supplementary Figure S6). In addition the evidence that LeEIX2 is initially accumulated as in the control (**Figure 5E**, 24–32 h), but is reduced at a later stage (**Figure 5E**, 40–48 h) allowed us to entertain the notion that this could be due to a biological interaction between the two proteins, and we proceeded to examine this further. Given that LeEIX2 is a plasma membrane protein, we hypothesize that SlPRA1A may be promoting LeEIX2 trafficking to the vacuole for degradation. To examine this hypothesis, we examined whether SlPRA1A overexpression enhanced the trafficking of LeEIX2 to the vacuole. We examined the co-localization of LeEIX2 and the FYVE MVB/LE marker 30 h post-agroinfiltration, when LeEIX2 expression is higher in a SlPRA1A overexpression background (see **Figure 5E**). We found that the co-localization of LeEIX2 and the late endosomal marker significantly increased in the presence of SlPRA1A, as compared to the control (**Figures 6A,B**). Thus, SlPRA1A induces LeEIX2 localization to late endosomes, the intermediary compartment for the vacuolar degradation pathway. To test whether LeEIX2 is degraded by the vacuolar degradation pathway, we used Concanamycin A (ConcA), which inhibits H^+^-ATPases, increasing vacuolar pH and inhibiting vacuolar proteases activity (Drose and Altendorf, 1997). LeEIX2 protein levels were detected by immunoblot (**Figure 6C**). Inhibiting vacuolar degradation allowed LeEIX2 to accumulate in a SlPRA1A overexpression background (**Figure 6C**), indicating that SlPRA1A may redirect LeEIX2 to the vacuole for degradation through the MVB/LE pathway.

### SlPRA1A Overexpression Decreases RLP-PRR Protein Level, But Does Not Affect RLK-PRR Protein Level

**FIGURE 7:**
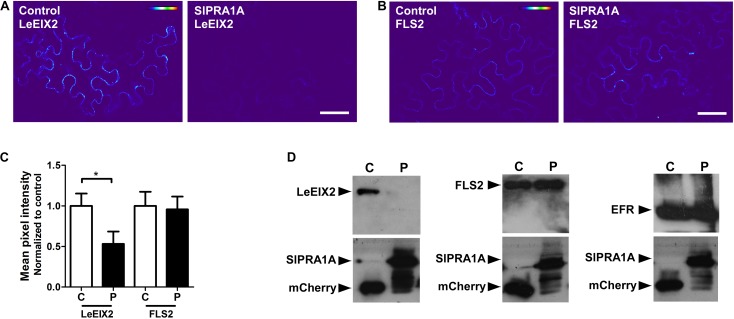
SlPRA1A does not induce the degradation of FLS a RLK-PRR. **(A,B)** Confocal microscopy images of *N. benthamiana* epidermal cells transiently expressing LeEIX2-GFP or FLS2 and free-mCherry (control) or SlPRA1A-mCherry. Representative images are shown. Scale bar 50 μm. **(A)** Heat map of LeEIX2-GFP (non-contrast adjustment) co-expressed with free-mCherry (Control), or SlPRA1A-mCherry. **(B)** Measurement of LeEIX2-GFP mean pixel intensity (*N* = 18). Data are represented as mean ± SD. Asterisks represent statistical significance (*p*-value ≤ 0.05) in Student’s *t*-test. **(C)** Heat map of FLS2-GFP (non-contrast adjustment) co-expressed with free-mCherry (Control), or SlPRA1A-mCherry. **(D)** Measurement of LeEIX2-GFP mean pixel intensity (*N* = 18). Data are represented as mean ± SD. Asterisks represent statistical significance (*p*-value ≤ 0.05) in Student’s *t*-test.

**FIGURE 8:**
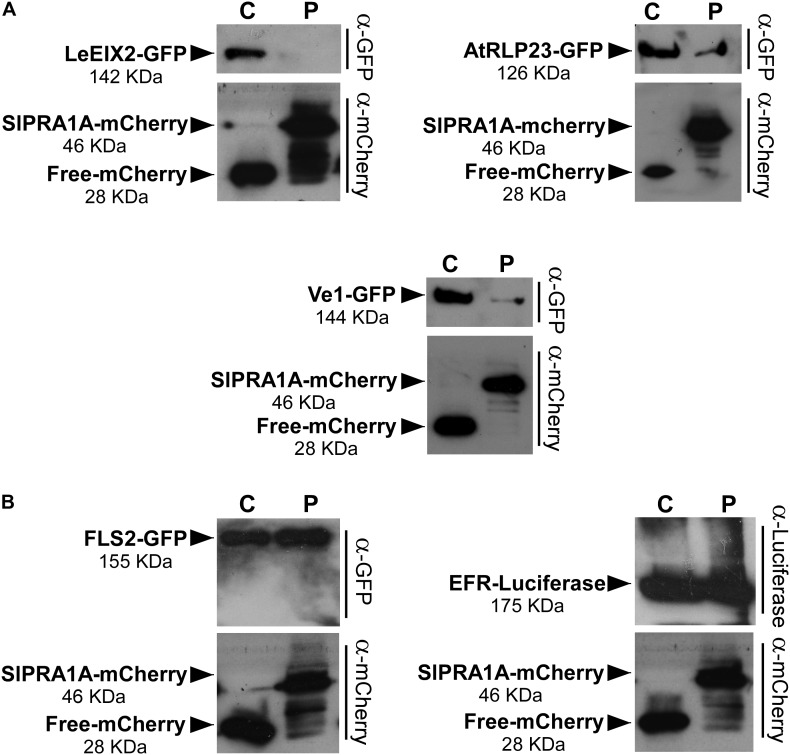
SlPRA1A induces degradation of RLPs, but not of RLKs. **(A)** Protein levels of the RLPs LeEIX2-GFP, AtRLP23-GFP, and Ve1-GFP in *N. benthamiana* leaves overexpressing free-mCherry (C) or SlPRA1A-mCherry (P) were detected by SDS-PAGE and western blot. **(B)** Protein levels of the RLKs FLS2-GFP and EFR-Luc in *N. benthamiana* leaves overexpressing free-mCherry (C) or SlPRA1A-mCherry (P) were detected by SDS-PAGE and western blot. The experiments were replicated four times independently. Representative images are shown.

Pattern recognition receptors are classified into two main groups according the presence or absence of a kinase domain, with PRRs containing a kinase domain designated receptor like kinases (RLK), whereas PRRs lacking a kinase domain are called RLP (Tang et al., 2017). Through live cell imaging we examined the effect of SlPRA1A overexpression on the well-known RLK PRR, FLS2 (Zipfel et al., 2006) (**Figure 7** and Supplementary Figure S7). Remarkably, SlPRA1A does not affect FLS2 protein levels opening up the possibility of a specific role for SlPRA1A in the degradation of certain PRRs. In order to address this assumption, we examined the effect of SlPRA1A on the protein levels of different RLPs and RLK proteins. Two other RLPs were tested, the *Verticillium* sp. race 1 receptor, Ve1, from *S. lycopersicum* (Kawchuk et al., 2001) and RLP23 from *A. thaliana* (Bi et al., 2014). SlPRA1A strongly enhanced the degradation of both RLP receptors (**Figure 8A**). In addition, the impact of SlPRA1A on the protein level of two RLK-type PRRs from *A. thaliana*, the EF-Tu receptor, EFR (Gomez-Gomez and Boller, 2000) and flagellin receptor, FLS2 (Zipfel et al., 2006) was tested. Interestingly, SlPRA1A overexpression did not alter the protein level of either RLK (**Figure 8B**), suggesting that the effect of SlPRA1A on PRR protein levels is specific for the RLP-type of PRRs, and is indeed biologically specific and not likely to be solely a result of protein overexpression or mislocalization.

## Discussion

In this work, we have identified and characterized the *S. lycopersicum* gene SlPRA1A, a novel interactor of the LeEIX2 RLP-PRR. SlPRA1A is a member of the prenylated acceptor of the RAB1 protein family. At the subcellular level, we observed a punctuate-reticular pattern of SlPRA1A in RabD2b compartments surrounding the ER in *N. benthamiana* and mainly in a punctuated pattern in *S. lycopersicum*. Interestingly, its closest *Arabidopsis thaliana* ortholog AtPRA1.F1 and AtPRA1.F4 also show a punctuated pattern, although in this case they are localized at Golgi compartments (Alvim Kamei et al., 2008; Lee et al., 2017). Remarkably, the size of the RabD2b compartments is altered by SlPRA1A, suggesting that SlPRA1A functions in these compartments. The mutant SlPRA1A^*N62T/Y 70A*^, does not change the size of RabD2b compartments indicating that this mutation produces an hypomorphic version of SlPRA1A. Considering the effect of SlPRA1A on RabD2b compartments, RabD2b or its orthologs in *S. lycopersicum* could be putative targets of the regulation mediated by SlPRA1A. Notably, RabD proteins have been involved in the regulation of cargo proteins exit from the ER in *A. thaliana*.

In this work we have showed that SlPRA1A and LeEIX2 can co-localize in a punctuated compartment ER-adjacent, presumably RabD2b compartments. Within these compartments SlPRA1A could regulate LeEIX2 trafficking. We observed that SlPRA1A overexpression decreases LeEIX2 protein levels and endosomal localization, which leads to a strong reduction in LeEIX2 signaling. The depletion of LeEIX2 at the plasma membrane could decrease LeEIX2 availability to bind EIX leading to the observed reduction in EIX-induced responses. As expected, SlPRA1A^*N62T/Y 70A*^ does not alter LeEIX2/EIX responses, although LeEIX2 protein level slightly decreased, indicating that the mutatated version of SlPRA1A does not generate a complete loss of function. Similarly, to our findings, in mammals a PRA1 protein has been shown to regulate receptor trafficking and signaling. LMP1, a viral TNF receptor, interacts with the human PRA1, impacting LMP1 trafficking and TNF signaling (Liu et al., 2006).

Our results suggest that the reduction of LeEIX2 protein level may result from protein degradation. There are reportedly two main degradation pathways for transmembrane proteins in plants. One is ER associated degradation (ERAD) taking place in the ER, which involves ubiquitin/proteasome mediated degradation of mutated/misfolded proteins, although participation of vacuolar degradation in ERAD has also been suggested (Pimpl et al., 2006; Su et al., 2011; Huttner and Strasser, 2012) The second degradation pathway for transmembrane proteins is the vacuolar degradation pathway (Muntz, 2007). Through this pathway, plasma membrane proteins are internalized to endosomes, then trafficked to MVB/LEs to finally reach the vacuole for degradation (Kleine-Vehn et al., 2008; Gu and Innes, 2012; Kolb et al., 2015). The vacuolar degradation pathway is considered specific and highly regulated by ubiquitination and by ligand interaction, in the case of receptors (Spallek et al., 2013; Smith et al., 2014; Zhuang et al., 2015). Our results suggest that SlPRA1A overexpression may mediate LeEIX2 degradation, possibly in a vacuole-dependent manner, and that SlPRA1A promotes LeEIX2 localization to MVB/LE, indicating that SlPRA1A may redirect LeEIX2 to MVB/LE and then to the vacuole, possibly for degradation. Additional PRRs, such as EFR and FLS2, were reported to be driven to the vacuole for degradation after elicitor perception, attenuating defense signaling (Lu et al., 2011; Spallek et al., 2013; Smith et al., 2014; Mbengue et al., 2016; Ortiz-Morea et al., 2016).

Recently, AtPRA1.F4 has been described as a Golgi membrane protein that regulates the trafficking of plasma membrane and vacuolar proteins (Lee et al., 2017). AtPRA1F4 overexpression inhibits the trafficking of plasma membrane and vacuolar proteins to their target compartment. Similar subcellular localization and functions have been described for AtPRA1.B6, which act as a negative regulator of trafficking to plasma membrane and vacuole (Heo et al., 2010; Lee M.H. et al. 2011, 2017). Interestingly, both PRA1 proteins from *A. thaliana* play a role as a negative regulator of trafficking to vacuole, in contrast to our data. Although in *O. sativa* OsPRA1 is localized at late endosomes and has been described as a positive regulator of trafficking toward the vacuole (Heo et al., 2010). Overexpression of a mutant version of this PRA1 protein impairs the trafficking of vacuolar proteins to their target compartment (Heo et al., 2010). Our findings showed that SlPRA1A is located in a RabD2b compartment which has properties of TGN/EE and Golgi, similarly to AtPRA1. F4 (Geldner et al., 2009; Peng et al., 2011), and SlPRA1A overexpression induce changes to the RabD2b compartment, suggesting that SlPRA1A function could be related with this compartment. Where SlPRA1A could alter the compartment morphology itself or change the intensity or place of RabD2b recruitment Additionally, SlPRA1 overexpression is able to redirect the trafficking of certain plasma membrane proteins, such as RLP-PRR, to the vacuole, in a similar way as OsPRA1. This could occur by the activation of trafficking pathways toward the vacuole, or by impairing the anterograde trafficking to the plasma membrane. It is important to mention that the effects induced by SlPRA1A overexpression could be due to a possible loss of relevant regulatory domains on the SlPRA1A tagged version, depletion of interactors for SlPRA1A, such as Rab proteins, and consequently, prevention of Rab-driven trafficking or compartment maturation. Therefore, the effect of SlPRA1A overexpression on trafficking could be due for an activation of trafficking pathways.

Interestingly, we show that SlPRA1A overexpression decreases the protein levels of other RLPs, such as AtRLP23 and Ve1, but does not change the protein level of FLS2 and EFR. This differential effect indicates that SlPRA1A may function as a regulator of trafficking and/or signaling of a particular type of RLPs, such as LeEIX2. This specificity suggests that SlPRA1A takes part in a trafficking machinery selectively regulating RLP trafficking. Selective trafficking has been described as the predominant trafficking model, where a specific trafficking machinery recognizes and sorts specific cargoes or transmembrane proteins, subsequently driving them to the target compartment (reviewed in Aridor and Traub, 2002; Gershlick et al., 2014; Veljanovski and Batoko, 2014). Therefore, our results open an intriguing venue to characterize the role of SlPRA1A role in RLP sorting, recognition, and trafficking from the ER-adjacent TGN/EE compartments toward the vacuole for degradation. Concomitantly, a SlPRA1A ortholog may regulate an analogous trafficking pathway for RLKs. Three main RABs are involved in regulation of plant vacuolar trafficking. RAB11, RAB5, and RAB7 act sequentially in the trafficking from MVB/LE toward the vacuole, a process involving several accessories, regulator proteins, and effectors, such as VTI11/12, SAND1/Mon1, CCZ1 complex and HOPS (Aridor and Traub, 2002; Niihama et al., 2009; Bottanelli et al., 2011; Ebine et al., 2014; Gershlick et al., 2014; Veljanovski and Batoko, 2014). Identifying the trafficking machinery involving SlPRA1A, and particularly determining whether RAB5 and RAB7 are targets of SlPRA1A, will be instrumental in deciphering SlPRA1A trafficking mechanisms and pathways. SlPRA1A is thus a new intriguing model candidate opening exciting research avenues into RLP-PRR specific trafficking, signaling and degradation pathways.

## Author Contributions

LP and ML-M: conceptualization. LP, ML-M, and SS: methodology. LP, ML-M, SS, and TM: investigation. LP: formal analysis and visualization. LP and AA: writing – original draft. ML-M; SS, MB, and AA: writing – review and editing. AA: funding acquisition.

## Conflict of Interest Statement

The authors declare that the research was conducted in the absence of any commercial or financial relationships that could be construed as a potential conflict of interest.
