# Supplementary figures and images for: Tomato Prenylated RAB Acceptor Protein 1 Modulates Trafficking and Degradation of the Pattern Recognition Receptor LeEIX2, Affecting the Innate Immune Response
Source: Front Plant Sci. 2018 Mar 1;9:257. doi: 10.3389/fpls.2018.00257 (PMC5838007; doi:10.3389/fpls.2018.00257)

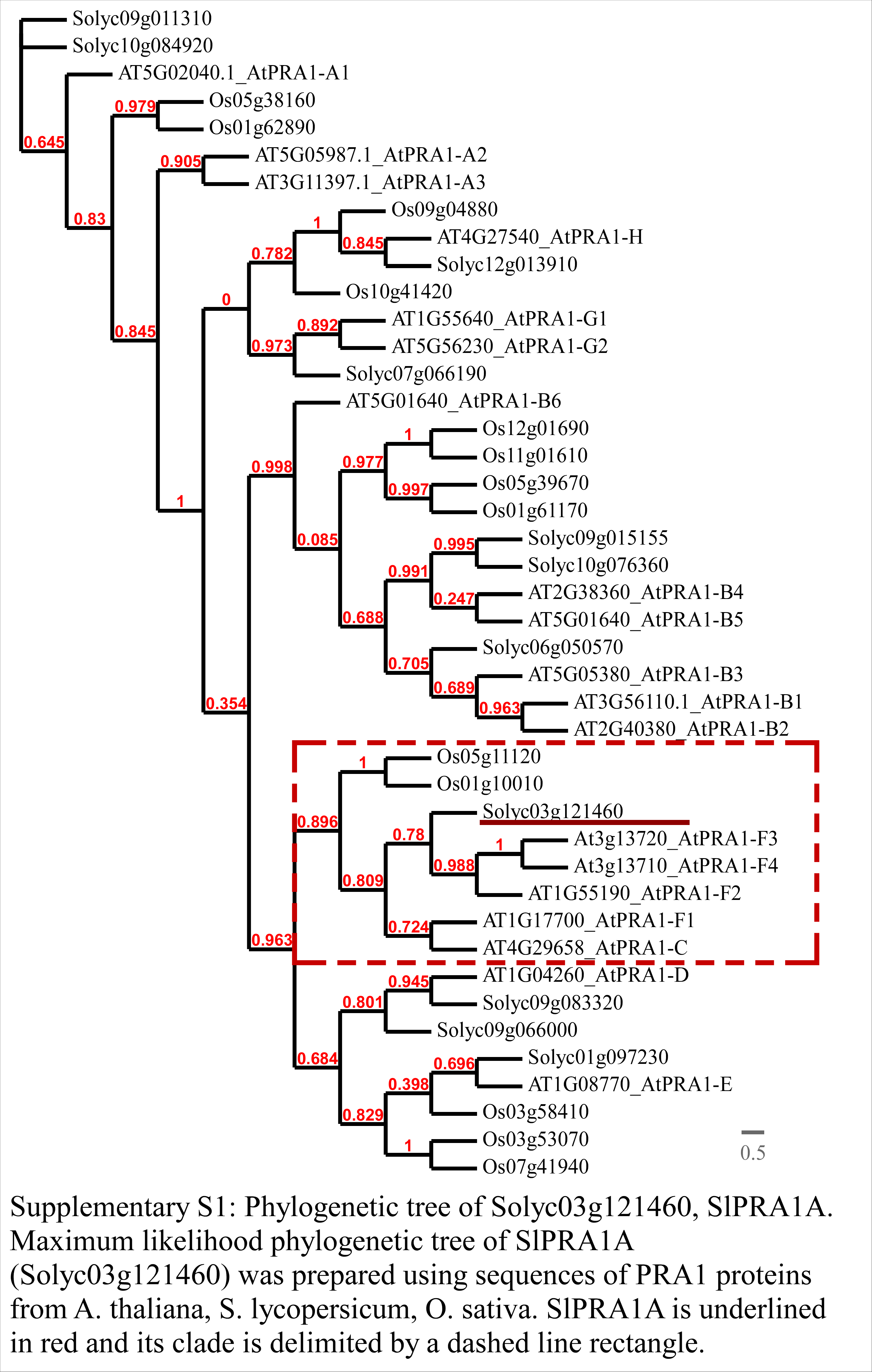

Supplement: Supplementary file 1 [file Image_1.TIF]

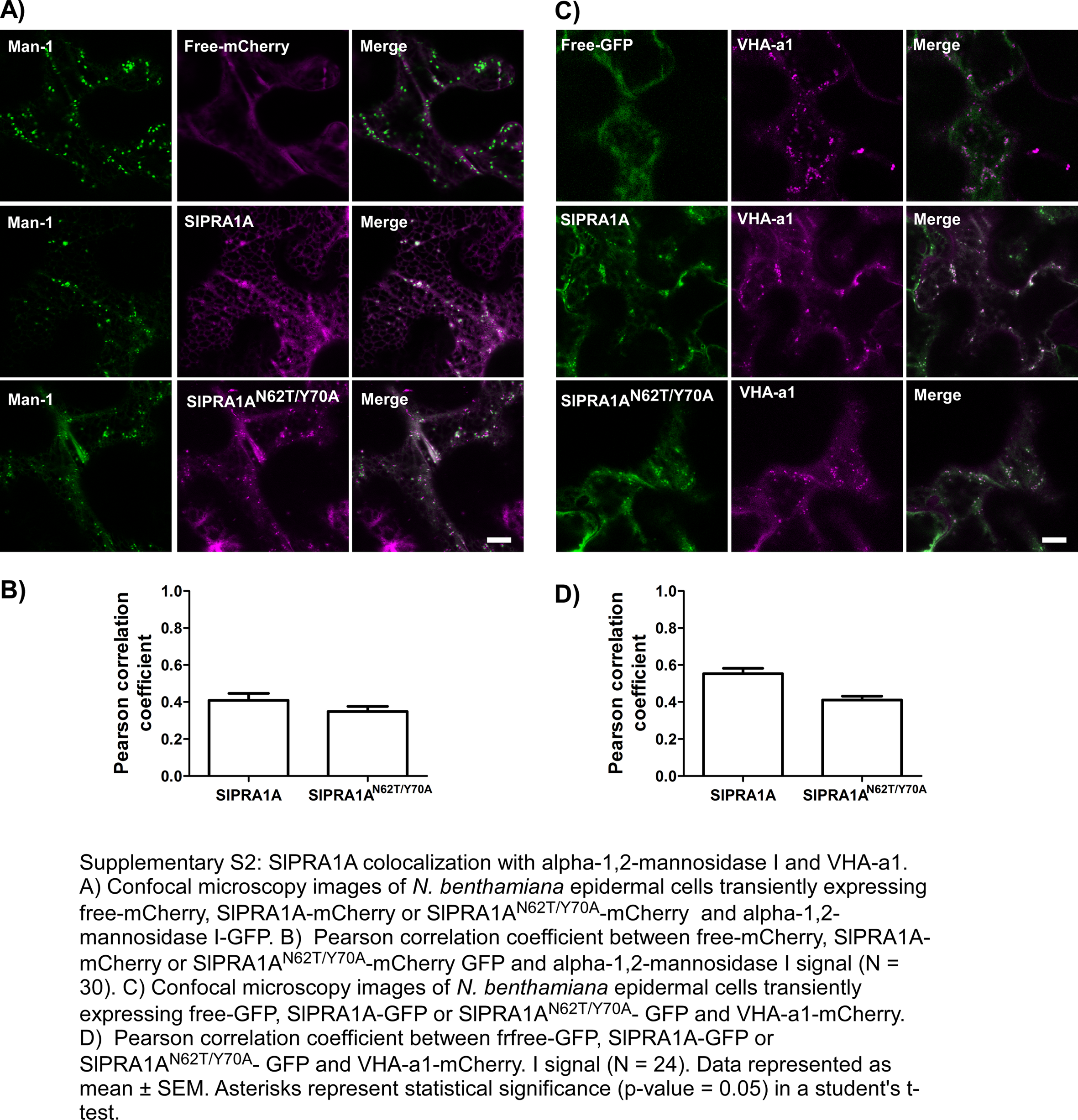

Supplement: Supplementary file 2 [file Image_2.TIF]

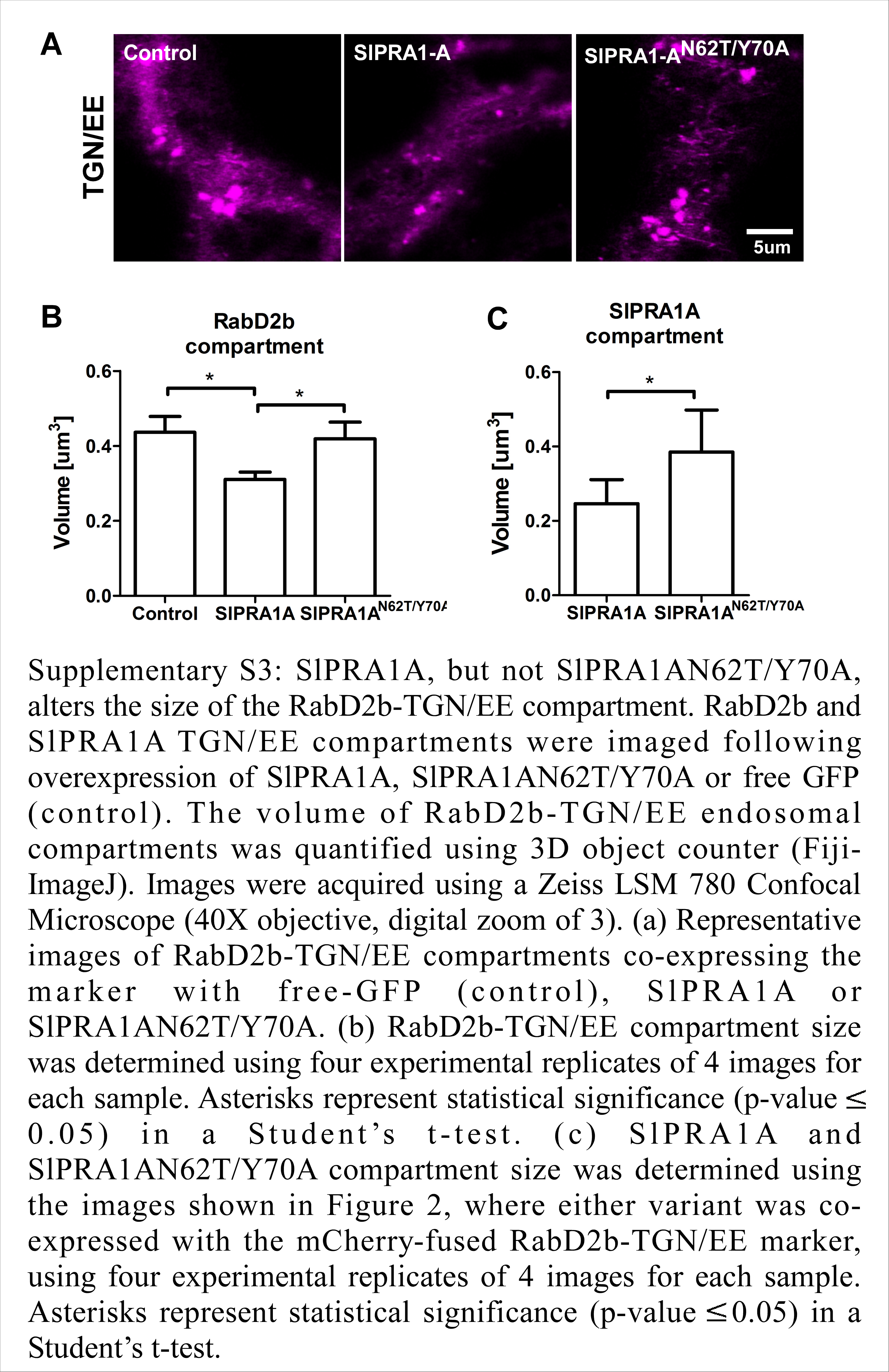

Supplement: Supplementary file 3 [file Image_3.TIF]

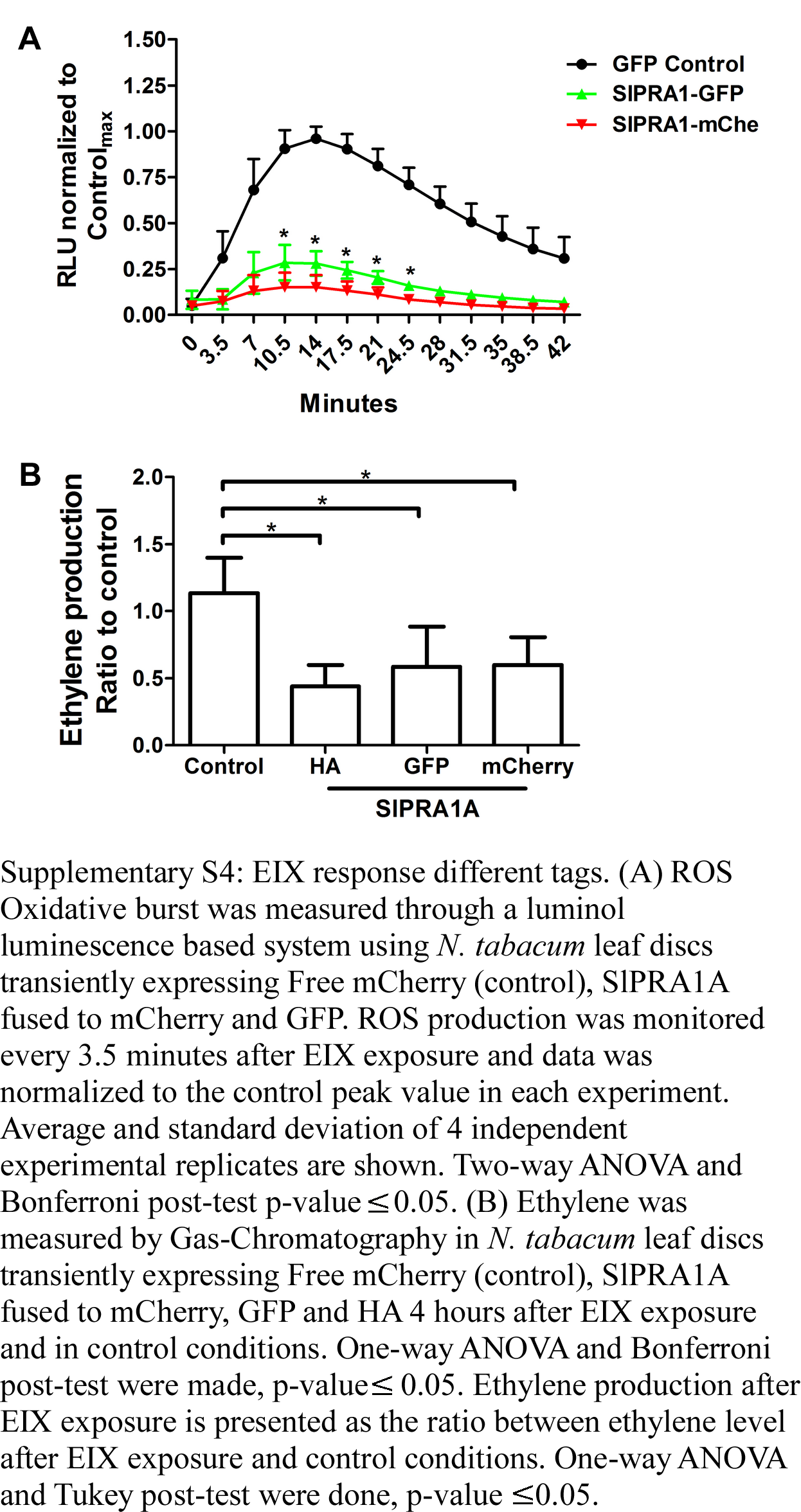

Supplement: Supplementary file 4 [file Image_4.TIF]

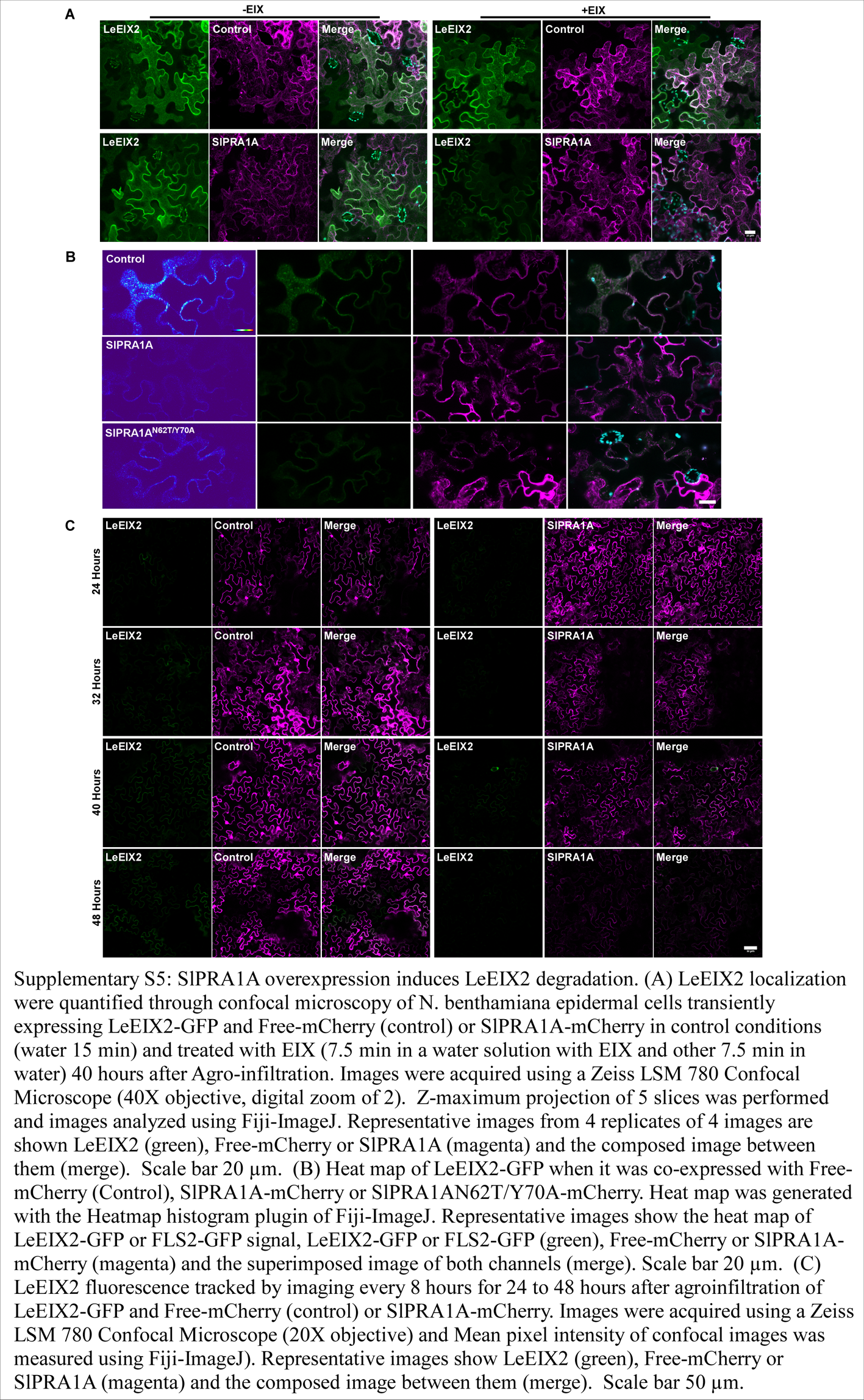

Supplement: Supplementary file 5 [file Image_5.TIF]

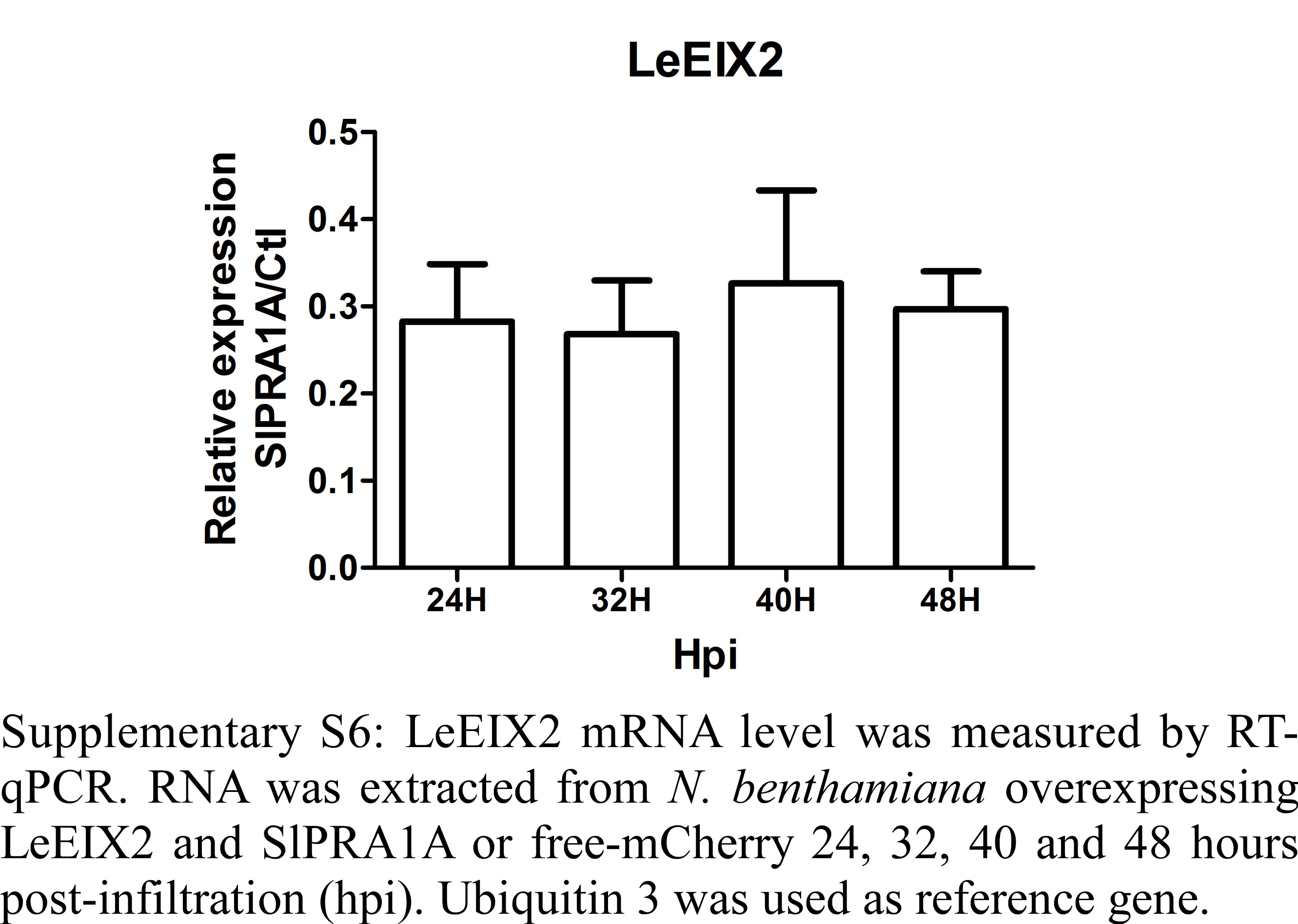

Supplement: Supplementary file 6 [file Image_6.TIF]

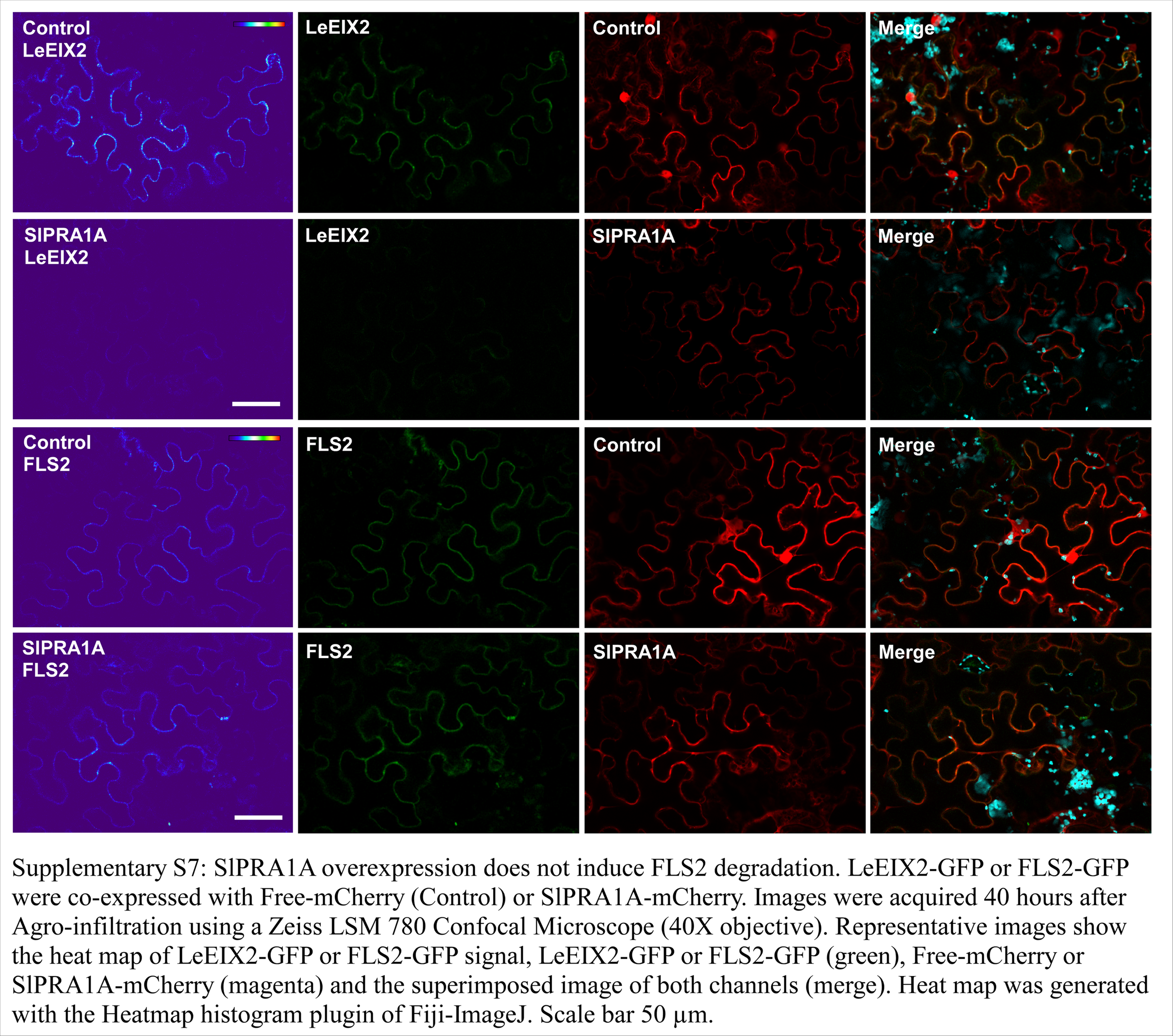

Supplement: Supplementary file 7 [file Image_7.TIF]
